# Bioaccessibility of Nickel and Cobalt Released from Occupationally Relevant Alloy and Metal Powders at Simulated Human Exposure Scenarios

**DOI:** 10.1093/annweh/wxaa042

**Published:** 2020-04-22

**Authors:** Xuying Wang, Inger Odnevall Wallinder, Yolanda Hedberg

**Affiliations:** KTH Royal Institute of Technology, School of Engineering Sciences in Chemistry, Biotechnology and Health, Department of Chemistry, Division of Surface and Corrosion Science, Drottning Kristinas v. 51, Stockholm, Sweden

**Keywords:** alloy powders, alloying effects, cobalt, corrosion, hazard classification, metal release, nickel, simulated human exposure

## Abstract

Nickel (Ni) and cobalt (Co) release from chromium-alloy powders (different stainless steels and a nickel-based Inconel alloy) compared with Ni and Co metal powders was investigated at simulated human exposure scenarios (ingestion, skin contact, and inhalation) between 2 and 168 h. All investigated powders consisted of particles sized within the respirable range. The powder particles and their surface reactivity were studied by means of nitrogen adsorption and electrochemical, spectroscopic (X-ray photoelectron spectroscopy and atomic absorption spectroscopy), light scattering, and microscopic techniques. The release of both Ni and Co was highest in the acidic and complexing fluids simulating the gastric environment and an inhalation scenario of small powders (artificial lysosomal fluid). Relatively high corrosion resistance and lower levels of released Ni and Co were observed in all fluids for all alloy powders compared with the corresponding pure metals. The extent of released metals was low for powders with a passive surface oxide. This study strongly emphasizes the importance of considering alloying effects in toxicological classification and/or regulation of Ni and Co in alloys and metals.

## Graphical Abstract

**Figure:**
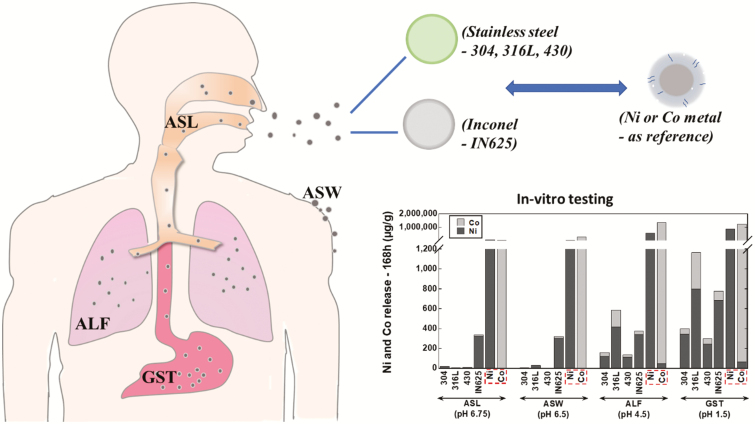

## Introduction

Humans are daily exposed to metal-containing particles via air pollution, and emissions from e.g. combustion and road traffic (Seaton *et al.*, 1995; Karlsson *et al.*, 2005). Exposure can also take place during various occupational activities, such as handling and disposal of metallic powder during e.g. 3D printing, processing of metallic products or during manufacture of metals and alloys (IARC, 1990, 2006; Mellin *et al.*, 2016). Human exposure to some metal-containing powders has shown to induce adverse health effects including inflammation and DNA damage, as well as pose carcinogenic and respiratory risks. Depending on the exposure setting, metallic powder particles can consist of metal compounds and/or be in their metallic forms. Nickel (Ni) and cobalt (Co) are two metals to consider in occupational health risk assessment. Recently, an occupational exposure limit (OEL) of 0.005 mg m^−3^ for respirable dust (0.03 mg m^−3^ for inhalable dust) for Ni metal and its compounds was recommended (RAC, 2018). This was followed by OEL values of 0.05 mg m^−3^ Ni (inhalable fraction) and 0.01 mg m^−3^ (respirable fraction) recommended by the European Commission Advisory Committee on Safety and Health at Work in 2019 (EC, 2019), which most probably will be in force within one of the next revisions of the Carcinogens and Mutagens Directive 2004/37/EC. The OEL for inhalable Co metal is 0.04 mg m^−3^ (ECHA, 2020a).

Ni metal powder (particle diameter <1 mm) is according to the current Classification, Labelling and Packaging (CLP) regulation classified for Skin Sens.1 (may cause an allergic skin reaction), STOT RE 1 (causes damage to organs), and Carc. 2 (suspected of causing cancer) hazards (ECHA, 2008a). No specific classification exists for Co powder, but Co metal is likewise classified as Skin Sens.1 and Resp. Sens. 1B (may cause asthma or breathing difficulties if inhaled) (ECHA, 2008b). Co metal will possibly be classified as Carc. 1B (may cause cancer), Muta. 2 (suspected of causing genetic defects), and Repr. 1B (presumed human reproductive toxicant) (RAC, 2017). Deliberations within an Expert Group (CARACAL) on the suitability of the classification methodology for metals have resulted in the proposal of a generic concentration limit (GCL) of 0.1 wt.% Co (bulk mass content) to replace the initially set limit of 0.01 wt.% Co (ECHA, 2017; RAC, 2017).

Skin contact (dermal), ingestion (oral), and/or inhalation are likely exposure routes for human exposure to metallic particles (Nordberg *et al.*, 2014). Such exposures need to consider factors such as particle size and size distribution. Particles with a diameter less than 100 µm are considered inhalable, which means that they may enter the respiratory tract via the nose and/or the mouth. Smaller particles (sized less than 11 µm) are defined as thoracic particles able to pass the larynx, and respirable particles (sized less than 5 µm) may reach the gas exchange region of the deep lung, possibly causing an inflammatory response (Ogden, 1992; ISO, 1995). Although potential health issues related to human exposure to metallic particles and prevailing mechanisms related to particle dissolution and metal release in contact with the human body are still relatively unexplored, recent studies suggest an evident correlation between the release of metals from the particles and their toxic potency for different endpoints (Ortega *et al.*, 2014; Cappellini *et al.*, 2018). *In vitro* dissolution investigations of metallic powder particles in synthetic body fluids (bioelution/bioaccessibility testing) have been conducted earlier (e.g. Hamel *et al.*, 1998; Voutsa and Samara, 2002; Stopford *et al.*, 2003; Twining *et al.*, 2005) and in recent years (e.g. Henderson *et al.*, 2012; Hillwalker and Anderson, 2014; Hedberg *et al.*, 2016; Kastury *et al.*, 2017; Heim *et al.*, 2020), elaborating reliable and reproductive *in vitro* test methods that have been applied to a range of metallic powder particles with the aim to generate short- or long-term quantitative metal release data used for hazard identification, classification, grouping, and read-across. Most of these studies have focussed on metal release (mainly of Cr, Fe, Ni, and other alloy constituents) from various alloy powders, such as ferrochromium- and nickel-containing alloys of specific relevance to exposure scenarios such as inhalation, ingestion, and skin contact. However, investigations on both Ni and Co release from stainless steels and nickel-based alloy powders are very rare compared with their widespread use (Baumers *et al.*, 2010; Li *et al.*, 2010). Apart from investigating the roles of pH, metal complexation capacity, and constituents of the test fluids on the metal release process, all these studies highlight the importance of the surface characteristics of metallic powder particles, especially the surface oxide composition and its electrochemical/chemical stability.

The aims of this study were therefore 2-fold: (i) to quantify the extent of Ni and Co release from alloy powder particles of relevance for several occupational settings and different exposure scenarios compared with the behaviour of Ni and Co metal powders, and (ii) to investigate and compare the surface reactivity of the different powders and its relation to the extent of Ni and Co release. This has been accomplished by kinetic *in vitro* bioelution testing (from 2 up to 168 h) using a recent elaborated test method for powders (Wang *et al.*, 2019) in fluids simulating ingestion (artificial gastric fluid—GST and artificial saliva—ASL), skin contact (artificial sweat—ASW), and inhalation (artificial lysosomal fluid—ALF) exposure scenarios. A multi-analytical approach was undertaken to characterize the powder particles and their surface reactivity, including several electrochemical, chemical, and surface analytical methods.

## Materials and methods

### Test materials and artificial biological media

The test materials include three stainless steel grade powders (AISI 304, 316L, 430), one nickel-based powder (Inconel 625—IN625), and two metal powders (Ni, Co), Table 1. Stainless steel is classified for causing respiratory irritation (STOT SE 3) under the CLP regulation (ECHA, 2020b), whereas Inconel 625 is currently without specific classification. The investigated alloy powders were supplied via Team Stainless, a cooperation between the International Stainless Steel Forum, the European Steel Association, the Nickel Institute, the International Chromium Development Association, the International Nickel Study Group, the International Molybdenum Association, and the ‘Stahlschrottverband in Deutschland’ (Germany).

**Table 1.** Nominal bulk composition (wt.%, based on supplier information) of investigated alloy (stainless steels 304, 316L, 430, and the nickel-based alloy IN625) and metal powders (Ni and Co).

Grade	EN number	C	Mn	Ni	Cr	Mo	S	Co	Fe
304	1.4301	0.02	1.44	9.4	18.8	N/A	0.008	0.07	69.6
316L	1.4404	0.01	0.97	10.5	16.6	2.2	0.004	0.06	69.0
430	1.4016	0.02	0.52	0.11	17.1	N/A	0.004	0.03	81.8
IN625	2.4856	0.01	0.24	63.7	21.2	9.1	0.006	0.01	2.2
Ni	Ni	N/A	N/A	99.99	N/A	N/A	N/A	0.012	N/A
Co	Co	0.033	0.002	0.003	N/A	N/A	0.001	98.7	N/A

N/A, no data available.

The extent of metal release from the alloy and metal powders was determined in four synthetic biological fluids simulating skin contact (ASW), inhalation (ALF), and ingestion (ASL and GST). The chemical compositions of each fluid, as well as their preparation details, are given in Supplementary Table S1 and Section S1.1, available at *Annals of Work Exposures and Health* online edition. The justification of the fluid compositions for these exposure endpoints has been reported previously (Stopford *et al.*, 2003; CEN, 2015; Hedberg *et al.*, 2016; Hedberg and Odnevall Wallinder, 2016). The fluids in this study were considered appropriate to, at least to some extent, mimic relevant human exposure routes even though they only simulate the physiological conditions to a limited extent. However, relative *in vitro* findings in the synthetic biological fluids can nevertheless provide useful and comparable information of relevance for *in vivo* conditions.

### Exposure procedure and metal release analysis

For each test item, the exposure procedure was followed by an elaborated method conducted on powder samples (Wang *et al.*, 2019). The powder samples were prepared with a 0.1 g l^−1^ loading (5 mg powder in 50 ml fluid). The exposure temperature was set at 37 ± 1°C (in the case of ASW: 30 ± 1°C), and the exposure periods to 4 and 168 h for all powders, and in addition 2, 8, and 24 h for the 316L powder. The short time periods are somewhat relevant to the inhalation/ingestion scenario, while the 168 h exposure time was only chosen to match the time period specified for the standard artificial sweat test EN1811 (CEN, 2015).

The solution samples were analysed for the total amount of released Ni and Co by means of atomic absorption spectroscopy with graphite furnace (GF-AAS) or flame (AAS) using a Perkin Elmer AA800 analyst instrument. All details are given in Supplementary Material (Section S1.2), available at *Annals of Work Exposures and Health* online edition. All reported data were calculated based on the mean value of triplicate sample concentrations of each powder with the respective blank sample concentration subtracted. Reported released amounts of metals, expressed in µg g^−1^, are based on blank-corrected concentrations of released metals (µg l^−1^) multiplied by the solution volume (l) and divided by the initial powder sample weight (g).

### Particle and surface characterization

#### Scanning electron microscopy

The surface morphology was characterized by means of scanning electron microscopy (FEI XL30 SEM and INCA software) with up to 100 000 times magnification using secondary electrons operating at a voltage of 20 kV. The powders were fixed on carbon tape to avoid their dispersion into the chamber and to ensure the best possible electrical conduction.

#### Brunauer–Emmett–Teller method

Specific surface areas (m^2^ g^−1^) of the powders at dry conditions were estimated using the Brunauer–Emmett–Teller (BET) method using a Micromeritics Gemini V analytical instrument (measured by Sandvik Heating Technology AB, Sweden). The measurement is based on the adsorbed amount of nitrogen gas at cryogenic conditions and was conducted at five different relative pressures for each powder.

#### Static light scattering and electrophoretic mobility measurements

Particle size distributions of the powders were determined in ASW, ASL, and ALF, respectively, using static light scattering (Malvern Mastersizer 3000 instrument). Instrumental limitations prohibited measurements in GST due to its low pH. The particle size was plotted against the volume percentage. Triplicate measurements were performed for each powder. More details are given in Supplementary Material (Section S1.3), available at *Annals of Work Exposures and Health* online edition.

Electrophoretic mobility measurements were conducted to estimate zeta potential values (using the Smoluchowski method) in 10 mM NaCl (pH 5.6) using a Malvern Zetasizer Ver. 7.11 (Uppsala, Sweden). Six measurements were performed at 25°C. The same input values of the refractive index as for measurements with static light scattering were used.

#### X-ray photoelectron spectroscopy

The average surface composition of the unexposed powder particles was analysed by means of X-ray photoelectron spectroscopy (XPS, UltraDLD spectrometer, Kratos Analytical) using a monochromatic Al X-ray source (150 W) on areas sized 700 × 300 µm^2^. Observed elements in the outermost surface oxide (approx. 5 nm) of the powders were analysed at two different areas and acquired with high resolution (20 eV pass energy) for Cr 2p, Ni 2p, Fe 2p, and Mn 2p. Binding energy correction was made using the C 1s adventitious carbon contamination peak at 285.0 eV.

### Electrochemical measurements

A paraffin-impregnated graphite electrode (PIGE), used as working electrode, has previously been shown sufficient to provide electrical conductance to powders and nanoparticles, and a very low background current compared with alternative working electrodes suitable for powders (Doménech *et al.*, 2011; Hedberg *et al.*, 2012a). Open circuit potentials (OCPs) of all powders were determined in all fluids using a PARSTAT MC Multichannel Potentiostat (Princeton Applied Research) equipped with a VersaStudio software, an Ag/AgCl saturated KCl electrode as reference electrode, and a platinum wire as counter electrode. Prior to the measurements, the unexposed powder (approx. 5 mg) was immobilized on the PIGE (diameter of 5 mm), pre-grinded with 1200 grit SiC paper, cleaned with ethanol and ultrapure water, and shortly heated before the powders were pressed onto its surface. Although it is impossible to exactly define the surface area and/or mass of the attached powders, each powder covered a surface area of 0.20 cm^2^. Special care was undertaken to ensure that this surface area was reached as exactly as possible and not exceeded. Loosely attached particles were manually shaken off. The OCP of each powder was monitored for 1 h at room temperature, and at least two replicates were performed for each powder and fluid.

To investigate the corrosion resistance of the different powders in the various fluids, potentiodynamic polarization measurements were carried out after stabilization of the OCP for 1 h. During polarization, the potential was swept from −0.2 to 1 V versus OCP at a scan rate of 1 mV s^−1^. More details are given in Section S1.4 in Supplementary Material, available at *Annals of Work Exposures and Health* online edition.

Cyclic voltammetry measurements were also conducted for surface speciation analysis. Details are given in Section S1.5 in Supplementary Material, available at *Annals of Work Exposures and Health* online edition.

## Results and discussions

### Particle and surface characterization

#### Particle size, size distribution, and morphology

**Figure 1.**
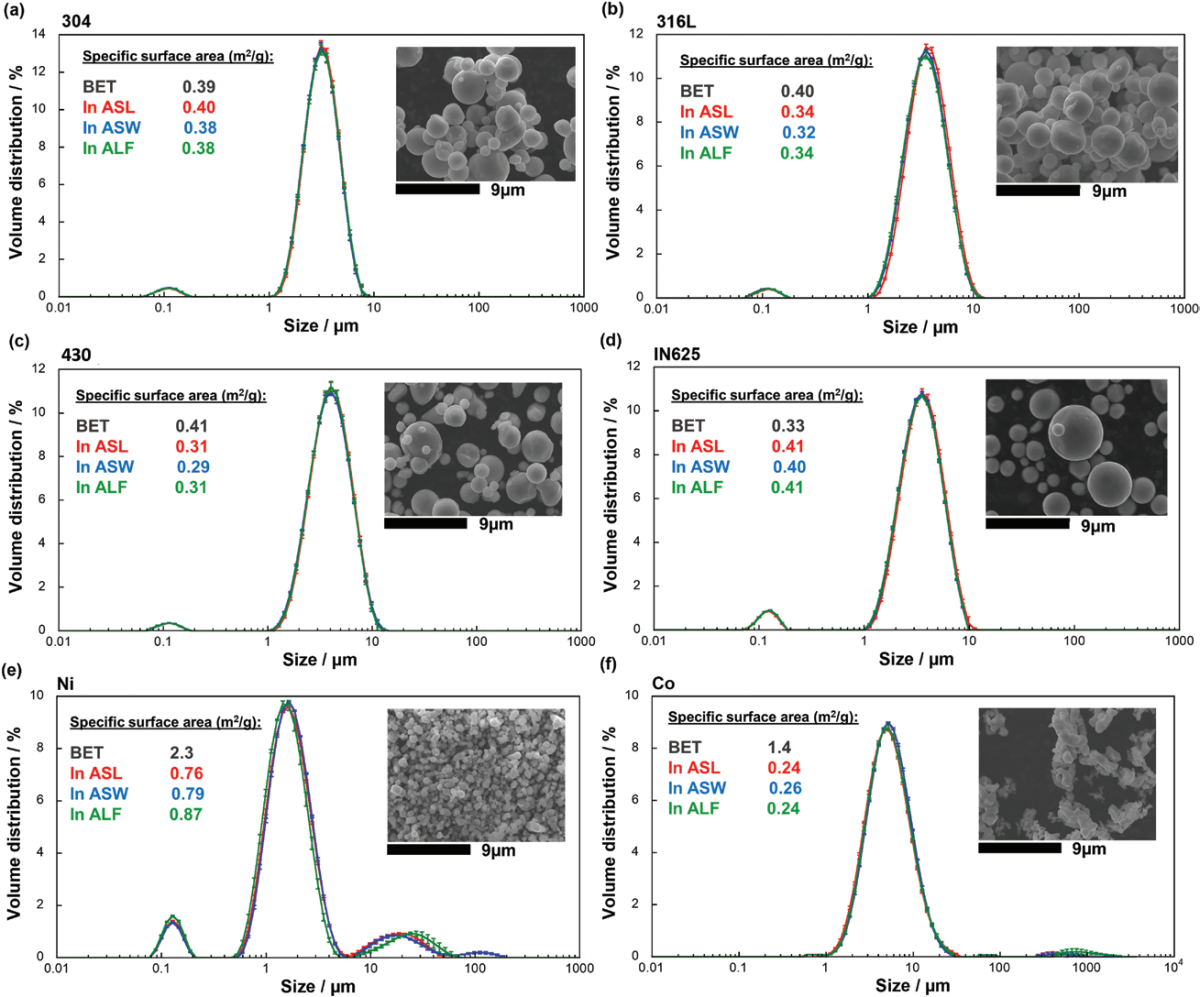
Particle size distribution (by volume) and calculated specific surface area (m^2^ g^−1^) of the alloy and metal powders (a: 304, b: 316L, c: 430, d: IN625, e: Ni and f: Co) in ASL (pH 6.75), ASW (pH 6.5), and ALF (pH 4.5) measured by means of static light scattering, and data on specific surface area (m^2^ g^−1^) at dry conditions based on nitrogen adsorption (BET). Average values of three measurements are shown with a standard deviation less than 10% (shown as error bars) in all cases for the size distribution values. The inset SEM images show the corresponding morphology of the particles at similar magnification for all powders (data partially based on Wang *et al.*, 2019 and included for comparison).

Fig. 1 summarizes information on powder particle morphology and their specific surface area at dry conditions (BET) and in the three synthetic body fluids (ASW, ASL, and ALF). The alloy powders (304, 316L, 430, and IN625) were all relatively spherical, and all powders were well within the respirable size range (<5 µm), confirming their relevance for investigations of the inhalation route. Judged from SEM imaging, the alloy powders showed a similar particle size, being larger compared with the Ni and Co metal powders. These observations are consistent with higher specific surface areas (BET) for the metal powders compared with the alloy powders. All powders showed similar and therefore comparable sizes in ASL, ASW, and ALF as judged from the size distribution measurements and the specific surface area calculations. However, agglomeration was evident within the fluids in the case of the Ni and Co metal powders, not observed at dry conditions. The metal powders showed an up to 5-fold difference in specific surface area in solution compared with dry conditions, which was not the case for the alloy powders. None of the powders was electrostatically stable in solution based on the zeta potential measurements (10 mM NaCl, pH 5.6) showing potentials close to 0 mV (data not shown). At least three of the powders (Ni, Co, and 430) were ferromagnetic, resulting in a strong driving force for agglomeration. It has previously been shown that also some small particles of austenitic stainless steel powders can be ferromagnetic for conditions with rapidly cooled manufactured powders (relevant for <4 µm gas-atomized austenitic stainless steel particles) (Hedberg *et al.*, 2011b).

#### Surface oxide

The composition of the outermost (about 5–10 nm) surface oxide of the unexposed powders measured by XPS are compiled in Table 2. No metallic signals were observed for the stainless steel powders (430, 316L, and 304), which implies an oxide thickness exceeding the information depth (>5–10 nm). The results suggest further the surface oxides to predominantly be composed of Mn(IV)-oxides (642.2 ± 0.2 and 644.2 ± 0.2 eV), Fe(II)/Fe(III)-oxides (711.2 ± 0.4 and 713.4 ± 0.4 eV), and Cr(III)-oxides (576.5 ± 0.2 and 578.4 ± 0.1 eV) (Biesinger *et al.*, 2011). In accordance with literature findings (Norell *et al.*, 1992; Linhardt, 1998; Hedberg *et al.*, 2013), oxidized Mn (indicative of Mn-species (IV), possibly MnO_2_) was strongly enriched (48–55 wt.% of the oxidized metals) within the outermost surface of the stainless steel particles compared with the bulk content (<1.4 wt.%, Table 1) followed by oxidized Fe and oxidized Cr. Surface enrichment of Mn is expected for inert-gas-atomized stainless steel particles and has previously been explained by the large affinity of Mn to oxygen (Hedberg *et al.*, 2012a). In the case of the Ni-based alloy powder (IN625), both oxidized Cr (575.9 ± 0.1, 577.1 ± 0.1, and 578.7 ± 0.03 eV) and Ni (855.8 ± 0.1 eV, possibly attributed to NiO/Ni(OH)_2_; Biesinger *et al.*, 2011) were observed, related to the high bulk content of both Ni (63.7 wt.%) and Cr (21.2 wt.%) of IN625. A detectable signal related to metallic Ni (852.8 ± 0.02 eV) indicated a relatively thin surface oxide (<5–10 nm). In all cases, oxidized Cr was present as Cr(III), findings in agreement with literature (Hedberg *et al.*, 2010, 2016; Hedberg and Odnevall Wallinder, 2016). Only oxidized Ni (main peaks at 854.0 ± 0.1 and 856.0 ± 0.1 eV) were observed in the case of the Ni metal powder, and only Co-oxides (781.1 ± 0.3 and 783.2 ± 0.1 eV) for the Co metal powder. The compositional findings correlated well with the cyclic voltammetry investigation (Supplementary Fig. S2 and Section S2.2, available at *Annals of Work Exposures and Health* online edition).

**Table 2.** Relative mass content (wt.%) of oxidized metals (Mn, Fe, Cr, Ni, and Co) within the outermost surface oxides of the unexposed powders measured by means of XPS. Average values and standard deviations reflect independent duplicate samples (each measured twice on different surface locations). XPS spectra of the surface oxides (Mn 2p, Fe 2p, Cr 2p, Ni 2p, and Co 2p) on the alloy and metal powders are presented in Supplementary Fig. S1, available at *Annals of Work Exposures and Health* online. <LOD, below limit of detection.

Grade	Mn	Fe	Cr	Ni	Co
430	48 ± 0.3	37 ± 1.4	15 ± 1.1	<LOD	<LOD
316L	49 ± 0.4	39 ± 2.4	12 ± 2.1	<LOD	<LOD
304	55 ± 1.6	32 ± 0.7	12 ± 0.9	<LOD	<LOD
IN625	<LOD	<LOD	64 ± 8.4	36 ± 8.4	<LOD
Ni	<LOD	<LOD	<LOD	100	<LOD
Co	<LOD	<LOD	<LOD	<LOD	100

### Corrosion resistance measurements

**Figure 2.**
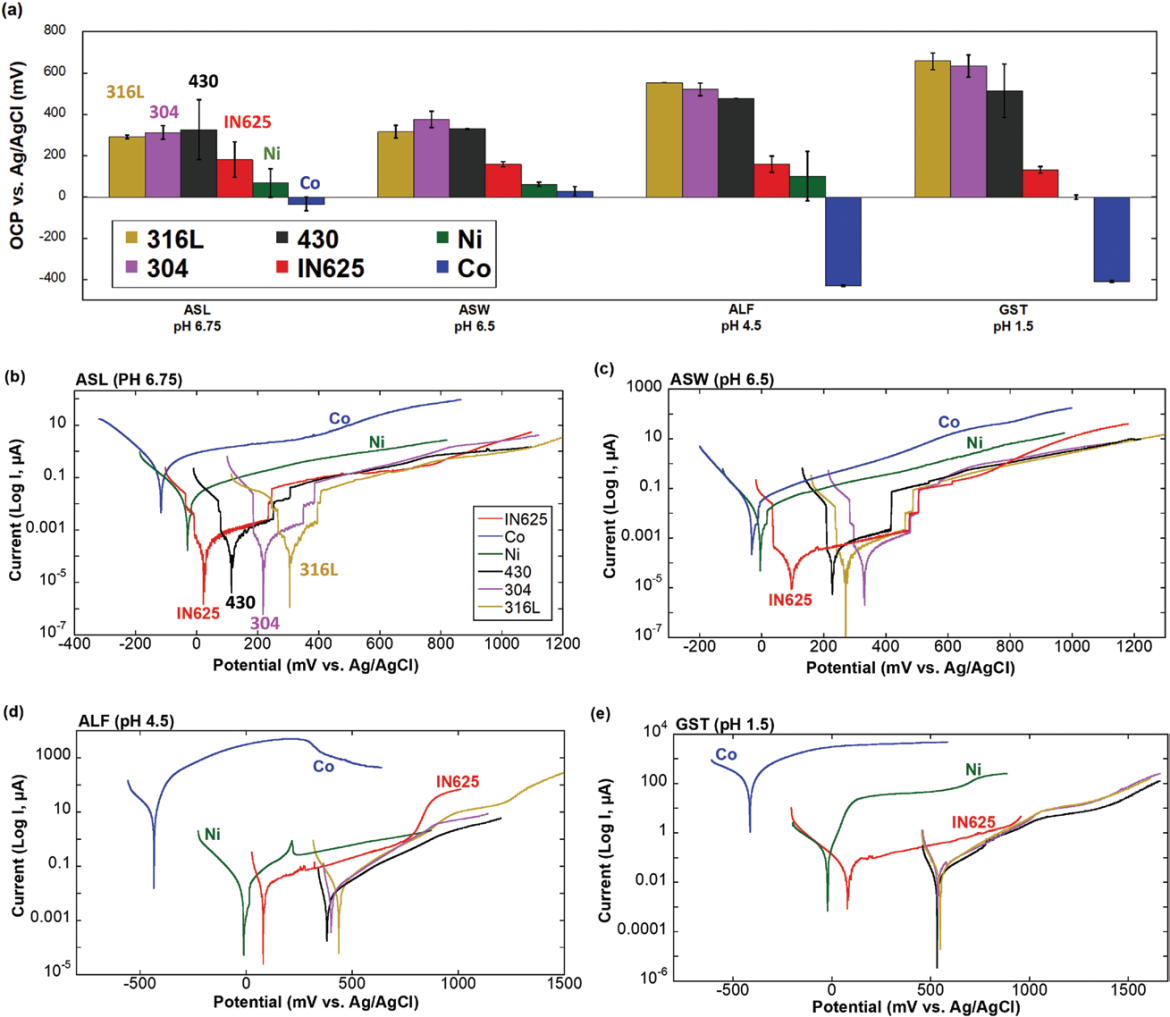
(a) Mean OCP values during 1 h for the alloy (316L, 304, 430, and IN625) and metal powders (Ni and Co) exposed in different synthetic biological fluids. The error bars represent the standard deviation between duplicate independent measurements. (b–e) Representative polarization curves of the investigated powders in each fluid after 1 h stabilization at OCP. *Note*: Different axis scales.

Fig. 2a shows the mean OCP values during 1 h of exposure for all powders and fluids. The stainless steel powders (316L, 304, 430) showed a significantly more positive OCP compared with the IN625 and the metal powders at all conditions, especially in the more acidic fluids (ALF—pH 4.5, GST—pH 1.5), which reflects the stronger passivity (lower corrosion tendency) of the surface oxide of the stainless steel powders. The relatively higher OCP of the stainless steel powders could furthermore be a result of the presence of Mn(III/IV)-phases (such as MnO_2_) (Dickinson *et al.*, 1996; Linhardt, 1997, 2004, 2010; Hedberg *et al.*, 2012a), that could catalyse the reduction of oxygen by chemical oxidation of Mn(III)-ions generated by the discharge of MnO_2_ (Cao *et al.*, 2003). The OCP of the stainless steel powders decreased with pH at a rate of ≈50 mV/pH, which is in accordance with the theoretical shift for one-electron transitions. As previously suggested (Hedberg *et al.*, 2012a), this implies that MnO_2_ is dissolved, or plays a minor role for the electrochemical reactions taking place in the most acidic fluids. The OCP of the IN625 powder was relatively independent of both test fluid and pH, and was slightly or substantially higher compared with the Ni and Co metals. This was most probably attributed to the Cr(III)-enriched surface oxide of IN625. The Co metal powder revealed the lowest OCP in all fluids, especially in ALF and GST, indicating that more severe active corrosion takes place in acidic solutions compared with neutral solutions.

Potentiodynamic polarization curves for one representative curve of each powder and solution after stabilization at OCP for 1 h are shown in Fig. 2b–e. The corrosion potential (*E*_corr_), corrosion current (*I*_corr_), and polarization resistance (*R*_p_) are reported for each powder in Supplementary Table S2, available at *Annals of Work Exposures and Health* online edition. The measurements were conducted for all powders attached onto the PIGE with the same powder-covered surface area (0.2 cm^2^) to enable comparison. Potentiodynamic curves of many, about 1 million, particles with a diameter of 5 µm that all behave as single cells are not directly comparable to massive surfaces (Hedberg *et al.*, 2012a,b). One or several solitary particles that become active would result in an increased current that would decrease after their complete dissolution, assuming that all other particles remain passive. Therefore, for powder electrochemistry, the potential can increase in several steps in the anodic range depending on which and how many particles that become active. In Fig. 2b–e, however, the current of all alloy powders remained lower than the corresponding current of the metal powders throughout the entire anodic range. This suggests no or minor active corrosion of the alloy particles. In the case of the pH-neutral fluids (Fig. 2b,c), the currents increased (in some cases in several steps) from ≈0.001 µA in the anodic range after the corrosion potential to approximately 0.1 µA. It remains unclear if these steps are connected to changes in measuring range of the potentiostat for such low currents or if it may be related to the activation of some particles within the powder. In the case of the more aggressive fluids (Fig. 2d,e), such steps were not observed due to a higher anodic current (0.1 µA). All stainless steel powders had, due to their passive Cr(III)-rich surface oxide (Hedberg *et al.*, 2010, 2012a, 2013), significantly lower corrosion susceptibility in all test fluids (lower *I*_corr_ and higher *R*_p_/*E*_corr_) compared with the metal powders, Fig. 2b–e and Supplementary Table S2, available at *Annals of Work Exposures and Health* online. The IN625 powder displayed a lower *E*_corr_ compared with the stainless steel powders (statistical significance *P* < 0.05 in most cases), partially related to the presence of Mn-oxides within the surface oxide of the stainless steel powders, but similar *I*_corr_ and *R*_p_ (*P* > 0.1 in most cases) in ASL, ASW, and ALF, Fig. 2b–d and Supplementary Table S2, available at *Annals of Work Exposures and Health* online. In GST, the most aggressive fluid (pH 1.5), the difference between the IN625 and the stainless steel powders was pronounced with a higher *I*_corr_ and lower *E*_corr_ for the IN625 powder. The metal powders (Ni and Co), in particular Co, showed a higher corrosion susceptibility illustrated by a significantly higher *I*_corr_ (reaching 160 µA in GST, 4000-fold higher than observed for 316L) and accordingly, a lower *R*_p_ in all fluids indicative of active corrosion. In ALF, the very high corrosion of the Co metal powder resulted in a decreased current at high potentials due to complete dissolution of a large number of powder particles, Fig. 2d. However, the corrosion current was 10-fold higher in GST compared with ALF, without any signs of reduced current due to dissolution. The dissolution process of Co metal powder in ALF was hence most probably also induced by chemical reactions (related to the high complexation capacity of ALF (Hedberg *et al.*, 2011a). For all powders, a significantly lower corrosion resistance was observed in ALF (pH 4.5) and GST (pH 1.5) compared with the two pH-neutral fluids (ASW—pH 6.5 and ASL—pH 6.75). These observations are in accordance with the expected behaviour (Hedberg and Odnevall Wallinder, 2016).

### Ni and Co release from alloy and metal powders in synthetic body fluids

#### Ni release

**Figure 3.**
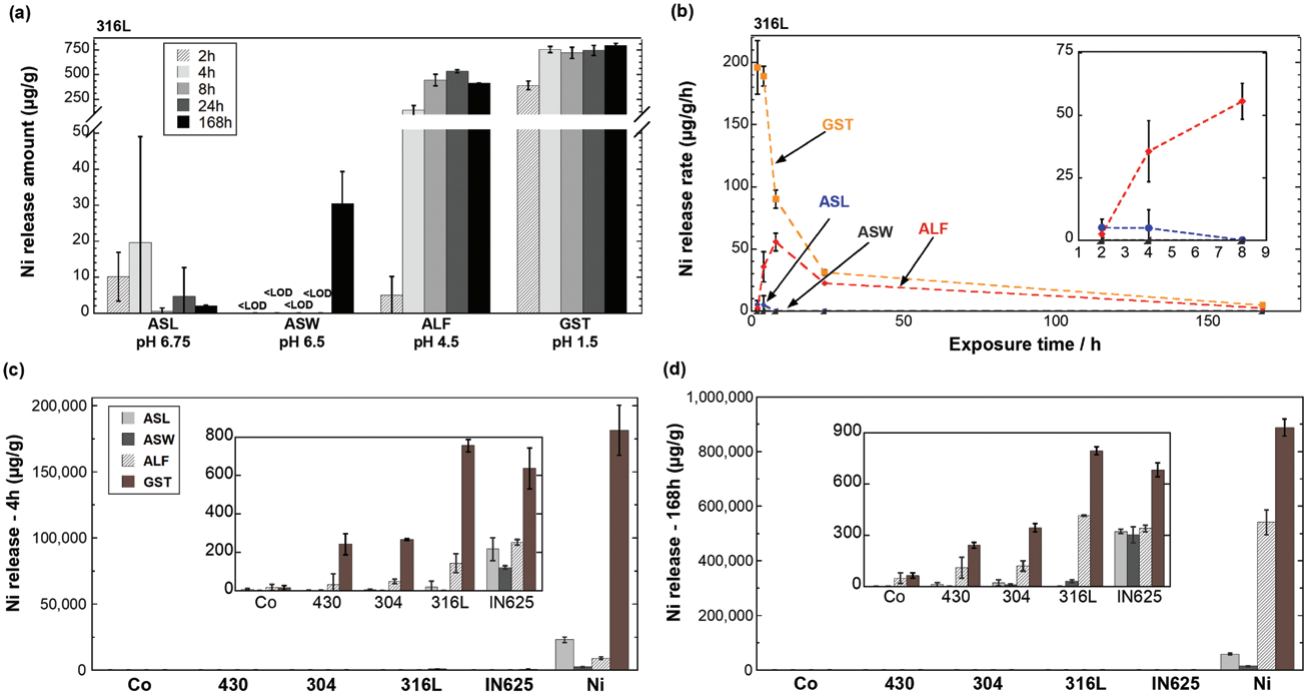
(a) Released amounts of Ni per particle mass (µg g^−1^) and (b) corresponding release rate (µg (g h)^−1^) from the stainless steel powder 316L immersed into ASL (pH 6.75), ASW (pH 6.5), ALF (pH 4.5), and GST (pH 1.5) for 2, 4, 8, 24, and 168 h (1 week). The inset graph in (b) shows a magnification of initial (first 8 h) release rates of Ni in ASW, ASL and ALF. Released amounts of Ni per powder mass (µg g^−1^) from Co metal powder and the different alloy powders (stainless steels—316L, 304, and 430; nickel-based alloy—IN625) immersed into ASL, ASW, ALF, and GST are presented after 4 h (c) and 168 h (d), with Ni metal powder as reference. The *x*-axis is ordered by an increasing nominal bulk content of Ni. The inset graphs in (c) and (d) show the magnification of released amounts of Ni. All data are shown as the average value of triplicate samples, and the error bars represent the standard deviation of triplicate samples. <LOD, below limit of detection. Corresponding raw data in Supplementary Tables S3–S7, available at *Annals of Work Exposures and Health* online.

Fig. 3a,b shows the amount of Ni released per particle mass (Fig. 3a) and per particle mass and time (h) (Fig. 3b) from the austenitic stainless steel powder 316L in the different synthetic body fluids after 2, 4, 8, 24, and 168 h. The release of Ni was highly pH/solution- and time-dependent, Fig. 3a. Most Ni release was observed in GST (pH 1.5) for all exposure periods and second highest in ALF (pH 4.5). In ASW (pH 6.5), the amount of released Ni was below the detection limit and first detectable after 168 h of exposure (30 ± 9 µg g^−1^). In ASL (pH 6.75), the amount of released Ni was slightly reduced with exposure time, possibly indicative of Ni precipitation from solution at that pH. Thermodynamic modelling of the Ni speciation in ASL at given conditions by the Joint Expert Speciation System (JESS) (May and Rowland, 2017) suggested solid γ-NiS to be the predominant phase (data not shown), which would explain the observed precipitation tendency. In the more acidic fluids ALF and GST, no obvious increase in Ni release was observed after 4 h of exposure, indicative of improved passive properties, findings in agreement with the OCP measurements (Fig. 2a) and also with previous findings showing the enrichment of Cr within the surface oxide of stainless steel in acidic solutions and ALF (Hedberg *et al.*, 2011a). Improved passive properties in the acidic fluids resulted in a strong reduction of the Ni release rate with time, Fig. 3b. However, the rate increased in ALF between 2 and 8 h before declining accordingly. Similar observations have previously been reported for FeCr particles and 316L powder (Midander *et al.*, 2010; Hedberg and Midander, 2014). These studies hypothesized that this effect is caused by the high content of citric acid in ALF, which results in a delayed complexation-induced metal release mechanism. This mechanism is particularly important for small (<5 µm) gas-atomized 316L powders, as the surface oxide to a greater extent is amorphous and hence more prone to complexation-induced release processes (Hedberg *et al.*, 2014; Hedberg and Midander, 2014).

Fig. 3c,d shows the amount of released Ni into the different fluids after 4 and 168 h for the four alloy powders (316L—10.5 wt.% Ni, 304L—9.4 wt.% Ni, 430L—0.11 wt.% Ni, and IN625—63.7 wt.% Ni) and the Ni and Co metal powders (Ni—99.999 wt.% Ni and Co—0.0027 wt.% Ni). All alloy powders released a lower amount of Ni (up to 20 000-fold) per particle mass compared with the Ni metal powder. As shown for 316L (Fig. 3a), all stainless steel powders (430, 304, and 316L) released significantly higher amounts of Ni in the acidic fluids (ALF and GST) as compared with the neutral fluids. However, this trend was less obvious for the IN625 powder, although this alloy also released the highest amount of Ni in GST compared with the other fluids. Compared with the 316L powder, the IN625 released a higher amount of Ni into the pH-neutral solutions—ASL and ASW, but a slightly lower amount into the most acidic fluid (GST). This is most probably related to the presence of oxidized Ni within the surface oxide of IN625, but not in the case of the stainless steel powders (Table 2). The influence of pH or solution composition of the test fluids on the Ni release from the alloy and metal powders was mostly in accordance with the electrochemical findings (Fig. 2). It is noteworthy that the observed amount of Ni release from the 316L powder in GST was higher than for the other stainless steel grades (304 and 430) and even for the IN625 powder. This may be a consequence of a higher extent of enriched Ni beneath the surface oxide of 316L (Hedberg *et al.*, 2012a), the presence of unstable Mn-phases within the surface oxide of coarse particles (>5 µm) or amorphous Mn-rich surface oxides of ultrafine (<4 µm) particles facilitating Ni release at acidic and/or complexing conditions (Hedberg and Midander, 2014).

#### Co release

**Figure 4.**
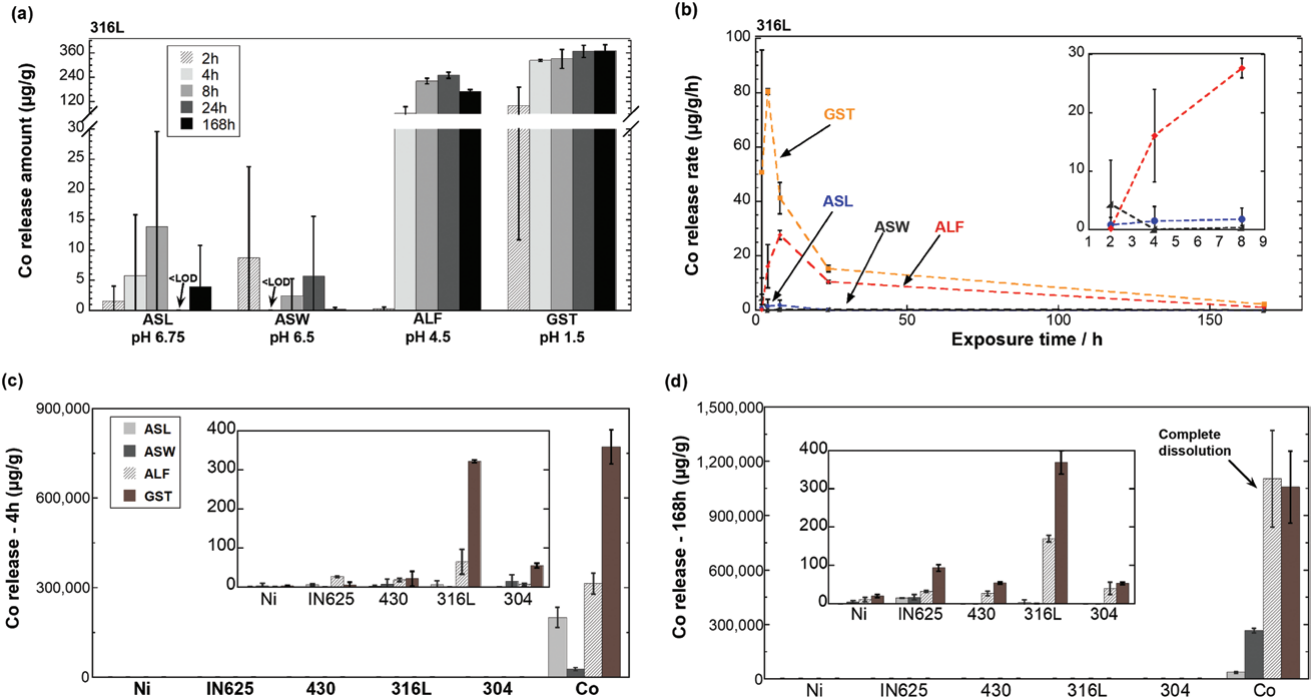
(a) Released amounts of Co per particle mass (µg g^−1^) and (b) corresponding release rate (µg (g h)^−1^) from the stainless steel powder 316L immersed into ASL (pH 6.75), ASW (pH 6.5), ALF (pH 4.5), and GST (pH 1.5) for 2, 4, 8, 24, and 168 h (1 week). The inset graph in (b) shows a magnification of initial (first 8 h) release rates of Co in ASW, ASL, and ALF. Released amounts of Co per powder mass (µg g^−1^) from Ni metal powder and different alloy powders (stainless steels—316L, 304, and 430; nickel-based alloy—IN625) immersed into ASL, ASW, ALF, and GST, for 4 h (c) and 168 h (d), respectively, with Co metal powder as reference. The *x*-axis is ordered by increasing nominal bulk content of Co. The inset graphs in (c) and (d) show the magnification of released amount of Co. All data are shown as the average value of triplicate samples, and the error bars represent the standard deviation of triplicate samples. <LOD, below limit of detection. Corresponding raw data in Supplementary Tables S7–S10, available at *Annals of Work Exposures and Health* online.

Fig. 4a,b shows corresponding results for the release of Co from the 316L powder. Consistent with Ni release findings, the highest released amount of Co was observed in GST for all time points, followed by ALF. No evident increase of released Co was observed in either GST or ALF after 4 h of exposure. In ASL, precipitation of released Co was obvious, as measured Co concentrations in solution decreased with time (after 8 h). JESS suggested the precipitation of solid β-CoS in ASL (data not shown). Release rates of Co from the 316L powder in GST, ALF, and ASL were in all cases initially low but increased with time reaching a maximum rate after 4 h in GST and after 8 h in both ALF and ASL, followed by declining rates to very low release rates after 168 h. This indicates, in addition to the complexation-induced delay of the release mechanisms discussed above, that Co is not as rapidly available for surface complexation as Ni, possibly due to its much lower bulk content. No clear trend was observed in ASW due to very low amounts of Co release and thereby large deviations (error bars) between triplicate samples.

Co release data from the stainless steel powders (316L—0.06 wt.% Co, 304L—0.07 wt.% Co, and 430L—0.03 wt.% Co), the Inconel powder (IN625—0.01 wt.% Co), and powders of Co metal (98.7 wt.% Co) and Ni metal (0.0027 wt.% Co) for reference are shown in Fig. 4c,d. The released amount of Co from each alloy was notably lower (up to 300 000-fold) than from the Co metal powder in all test fluids. The release of Co from the 316L powder in the more acidic fluids (ALF and GST) was substantially higher compared with the other stainless steel and the IN625 powders. This could be explained by the same reasons as the elevated Ni release, but the prevailing reason cannot be discerned. After the initial time period, almost no additional amount of Co was released from the stainless steel powders in either ALF or GST (equal amounts after 4 and 168 h), while there was a strongly increased amount of released Co for the IN625 powder in GST (from 5 µg g^−1^ after 4 h to 94 µg g^−1^ after 168 h). This was also reflected by the higher corrosion current for this powder in GST compared with the other alloys (Supplementary Table S2, available at *Annals of Work Exposures and Health* online).

### Relative bioaccessibility and aspects for hazard assessment

#### Ni release

Bioaccessibility is in this study defined as the released mass fraction of Ni or Co from the different powders into the synthetic fluids simulating different human exposure routes. A general definition is that the bioaccessible metal fraction can be available for absorption and possibly pose an adverse health hazard. The relative bioaccessibility of released Ni is calculated by the released amount of Ni (divided by the powder mass and Ni bulk content) from the alloy powders compared with corresponding amounts of released Ni from Ni metal powder exposed at parallel conditions (equation (1)).

$$\text{Relative bioaccessibility}=\frac{{\text{Released amount alloy}}_{\text{Co or Ni}}(\text{ ​​}\mu \text{​​ g/g})}{{\text{Bulk content alloy}}_{\text{Co or Ni}}(\text{wt}\%)}/\frac{{\text{Released amount metal}}_{\text{Co or Ni}}(\text{ ​​}\mu \text{​​ g/g})}{{\text{Bulk content metal}}_{\text{Co or Ni}}(\text{wt}\%)}$$ (1)

**Figure 5.**
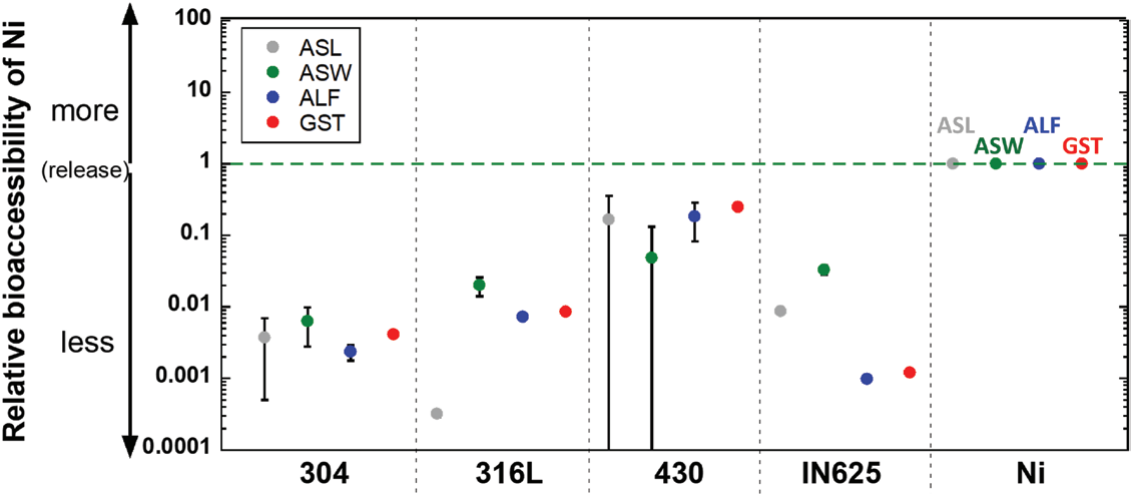
Calculated relative bioaccessibility of Ni released from all alloy and metal powders exposed under parallel conditions for 168 h (1 week). For example, a value of 0.01 means 100 times lower release of Ni per Ni alloy content (by mass) as compared with the Ni metal powder. Per definition, the relative bioaccessibility of the Ni metal powder equals 1, equation (1). All data are shown as the average value of triplicate samples, and the error bars represent the standard deviation of triplicate samples.

Fig. 5 shows the relative bioaccessibility of Ni after 168 h of exposure for the different fluids and alloys. Substantially lower relative bioaccessibility of Ni (ranging from 0.00032 to 0.25, i.e. 4–3000-fold lower than expected based on its bulk content) was observed for all alloy powders compared with the Ni metal powder. As discussed above, this is anticipated for corrosion-resistant alloys due to their passive surface characteristics. Similar findings were obtained calculating the relative bioaccessibility of Ni after 4 h, a time point possibly more relevant than 168 h for any actual exposure (Supplementary Fig. S3, available at *Annals of Work Exposures and Health* online edition). Most alloys showed a relative bioaccessibility being substantially less than 1, except for 430 in ALF and GST slightly exceeding 1.

REACH (Registration, Evaluation, Authorisation and Restriction of Chemicals) is the European chemical regulatory framework (first implemented in 2007) (EC, 2007) in which information on registered substances (including Ni and Co) related to e.g. toxicological endpoints are compiled for different exposure routes. Potential human hazards that may be induced by the exposure to metal particles depend not only on their physico-chemical characteristics but also on aspects including the exposure scenario and the dose.

Table 3 summarizes particle doses that would be required for the different powders to exceed toxicological endpoints stipulated for Ni in REACH. The derived no effect levels (DNELs) available are derived in different ways. For skin exposure, the DNEL of 0.035 mg cm^−2^ is derived from occluded patch testing (48 h) of 100% bioaccessible Ni sulphate on Ni-sensitized individuals, and further adjusted for the relative bioaccessibility of Ni ions released from Ni metal versus Ni sulphate in ASW added with an assessment factor of 2 to account for remaining uncertainty (ECHA, 2020c). Hence, this DNEL value was assumed to be the corresponding allowed maximum dose for the Ni metal powder in Table 3, whereas the surface area (cm^2^) applies to the skin area, not the powder surface area. With this value as a reference, and considering the difference in bioaccessibility between the Ni metal and the alloys, the corresponding maximum dose in Table 3 was calculated by multiplying the value for Ni metal with its Ni release (µg g^−1^) and divided by the Ni release from the alloy (µg g^−1^). These calculations gave at most a value of 16.8 (316L) and 1.7 (IN625) mg cm^−2^, which is approximately 50–500-fold higher doses compared with Ni metal powder. A similar assumption was made for the DNEL values for the inhalation route (for workers), with about 1000–2000-fold higher doses for the alloy powders compared with the Ni metal powder (0.05 mg m^−3^), Table 3. For the ingestion route, the alloy powder doses would be 250-fold higher, assuming a body weight of 70 kg, compared with the Ni metal powder (0.0042 g day^−1^), Table 3.

**Table 3.** Doses of the investigated powders (all alloys and metals) that would be required to exceed different reported toxicological endpoints for Ni relevant to skin contact, inhalation and ingestion exposure scenarios. Note that only 316L is included here as stainless steel representative due to its highest Ni release among the investigated stainless steel powders.

Investigated powder		Stainless steel	Ni-based alloy	Metal
		316L	IN625	Ni
Skin contact	Highest Ni release in ASW (µg g^−1^)^a^ (168 h)	30	303	14 362
	Toxicological endpoint	DNEL^b^ value: 0.035 mg cm^−2^ (ECHA, 2020c)^c^		
	Corresponding max. dose (mg cm^−2^)	16.8	1.7	0.035
Inhalation	Highest Ni release in ALF (µg g^−1^)^d^	434	304	541 566
	Toxicological endpoint	Workplace DNEL^b^ value: 0.05 mg m^−3^ (ECHA, 2020c)^e^		
	Corresponding max. dose (mg m^−3^)	62.4	89.1	0.05
Ingestion	Highest Ni release in ASL, GST (µg g^−1^)^f^	756	637	181 430
	Toxicological endpoint	DNEL^b^ value: 0.011 mg (kg bw)^−1^ day^−1^ (ECHA, 2020c)^g^		
	Corresponding max. dose (g day^−1^)^h^	1.0	1.2	0.0042

^*a*^The highest amounts of Ni release in ASW (worst-case) were after 168 h for all investigated powders.

^*b*^Derived no effect level.

^*c*^DNEL valid for both workers and the general population after long-term exposure. It is valid for Ni metal. The DNEL reflects the exposure needed to elicit a dermatitis response in already Ni-sensitive individuals.

^*d*^Highest Ni release in ALF (worst case) after 24 h (316L) or 168 h (IN625 and Ni) of exposure selected for the calculation even though 8 h of exposure is more relevant for workers.

^*e*^Reported value for workers, long-term exposure (systemic and local effects), referring to 8 h, inhalable aerosol fraction.

^*f*^Highest Ni release in GST compared with ASL (worst case) after 4 h of exposure. The time point was regarded relevant for gastrointestinal exposure.

^*g*^DNEL value reported for the general population, not for workers. bw, body weight.

^*h*^The calculation assumes a person with a 70 kg body weight.

Available assessments of Ni exposure at relevant occupational settings focus today mostly on the total metal fraction and do not either distinguish between reactive or less reactive powders. Exposure doses of 9 µg m^−3^ (median) total Ni, with the 95th percentile being as high as 460 µg m^−3^ have been reported for different German occupational settings (Kendzia *et al.*, 2017). Highest levels were observed in working environments of welders, metal sprayers, grinders, forging-press operators, and manufacturers of batteries and accumulators (Kendzia *et al.*, 2017). An important refinement for hazard assessment would be to consider the reactivity and amount of Ni release from the particles that the workers actually are exposed to, as these largely differ depending on the surface characteristics (Mei *et al.*, 2018).

#### Co release

**Figure 6.**
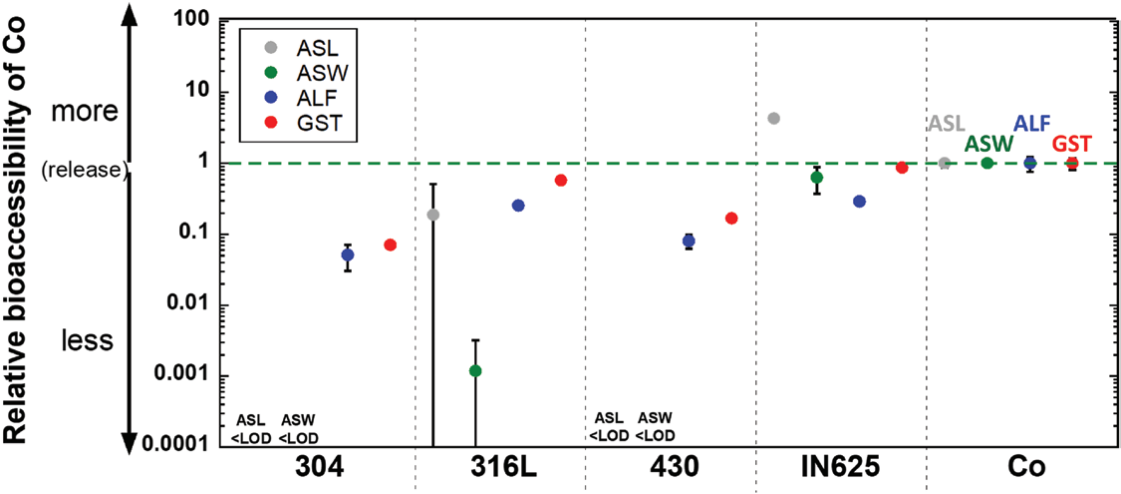
Relative bioaccessibility of Co from all alloy and metal powders exposed to the synthetic biological fluids for 168 h (1 week). For example, a value of 0.01 means 100 times lower release of Co per Co alloy content (by mass) compared with Co metal. Per definition, the relative bioaccessibility equals 1 for the Co metal powder, equation (1). All data are shown as the average value of triplicate samples, and the error bars represent the standard deviation of triplicate samples.

Fig. 6 summarizes the relative bioaccessibility of Co (equation (1)) from all alloy powders compared with Co metal exposed for 168 h in the four synthetic biological fluids. All alloy powders exhibited a relative bioaccessibility less than 1 (ranging from 0.042 to 0.75) in the most aggressive fluids ALF (pH 4.5) and GST (pH 1.5), meaning lower amounts (1.3–24-fold) of released Co when compared with their corresponding bulk content and compared with the release of Co from the Co metal powder. However, in the less aggressive fluids (ASL—pH 6.75 and ASW—pH 6.5), the relative bioaccessibility exceeded 1 after 168 h for the IN625 powder and was not possible to calculate for the 430 and 304 powders due to non-detectable amounts of released Co. This is possibly explained by the precipitation of released Co from solution resulting in low detectable amounts of Co after 1 week for the alloy powders and hence probably in underestimated released amounts in the case of the Co metal powder. Calculated relative bioaccessibility data for Co after 4 h are presented in Supplementary Fig. S4, available at *Annals of Work Exposures and Health* online edition. The results showed a relative bioaccessibility of less than 1 for most alloy powders and greater than 1 for 430 in ALF.

Table 4 presents required dose values of Co corresponding to toxicological endpoints calculated in a similar way as presented for Ni in Table 3. No DNEL value on systemic effects via the dermal route is reported in REACH. How and if the release of Co from the powders of this study relates to reported threshold values using patch testing for Co-allergic persons has recently been discussed (Wang *et al.*, 2019). None of the alloys of this study was at risk to exceed those threshold values, even at conditions of complete skin coverage. Following inhalation using the reported DNEL value for workers, the alloy powders would result in 4400–34 000-fold higher corresponding maximum doses compared with Co metal powder when considering their relative bioaccessibilities, Table 4. Following similar assumptions as given for Ni above and a body weight of 70 kg, the highest allowed daily maximum dose of the alloy powders would be 2400–150 000-fold higher for the alloy powders compared with the Co powder metal (0.0027 g day^−1^), Table 4.

**Table 4.** Doses of investigated powders (all alloys and metals) that would be required to exceed different reported toxicological endpoints for Co relevant to inhalation and ingestion exposure scenarios. Note that only 316L is included here as a stainless steel representative due to its highest Ni release among the investigated stainless steel powders.

Investigated powder		Stainless steel	Ni-based alloy	Metal
		316L	IN625	Co
Inhalation	Highest Co release in ALF (µg g^−1^)^a^	250	32	1 102 698
	Toxicological endpoint	Workplace DNEL^b^ value: 40 µg m^−3^ (ECHA, 2020a)^c^		
	Corresponding max. dose (µg m^−3^)	176 432	1 378 373	40
Ingestion	Highest Co release in ASL, GST (µg g^−1^)^d^	321	5	772 162
	Toxicological endpoint	DNEL value: 29.8 µg (kg bw) ^−1^ day^−1^ (ECHA, 2020a)^e^		
	Corresponding max. dose (g day^−1^)^f^	6.5	417.2	0.0027

^*a*^Co release in ALF after 168 h (for IN625 and Co) or after 24 h (for 316L), which is the highest release in all cases, selected even though 8 h of exposure is more relevant for workers.

^*b*^Derived no effect level.

^*c*^Reported value for workers, long-term exposure (local effects), referring to 8 h, inhalable aerosol fraction.

^*d*^Highest Co release in GST compared with ASL (worst case). 4 h of exposure selected relevant for gastrointestinal exposure.

^*e*^Body weight.

^*f*^Assuming a person with 70 kg body weight.

#### Implications on hazard assessment of alloy powders

This study strongly emphasizes the importance of considering alloying effects for toxicological classification and/or regulation of Ni and Co in alloys and metals. This study shows that the relative bioaccessibility can largely differ from 1, which suggests that this parameter, rather than the bulk metal content of alloys, should be used for toxicological classification/regulation. A positive example of considering chemical and material properties is the Nickel Directive, achemical directive for articles and items intended for skin contact in which the restriction limit is based on bioaccessibility testing of released Ni normalized to the exposed surface area (EC, 2007). The directive does however not cover any powders of relevance for occupational exposure. In the regulation of CLP of substances and mixtures (CLP Regulation), no distinction is made between mixtures and alloys. The recently discussed GCLs for Co in mixtures regarding the classification as carcinogen Carc. Cat. 1B (H350) are accordingly not differentiated for simple mixtures or alloys (ECHA, 2017; RAC, 2017). The use of bioaccessibility data to refine the classification of alloys is a possibility that remains, with the exception of the Nickel Directive, to be discussed and considered by regulatory authorities.

In this study, all alloy powders released lower amounts of Ni and Co when compared with the metal powders and when considering their corresponding bulk alloy contents. Alloying does not necessarily result in lower release of certain metals. Based on their known corrosion and physico-chemical properties, different metals and alloys could possibly in future be grouped into different classes based on their expected reactivity for a given exposure scenario. A low reactivity in one fluid does though not necessarily imply a low health hazard as this depends on the exposure route (e.g. ingestion or inhalation), the particle size and other physico-chemical characteristics of the powders (Oberdörster *et al.*, 2005).

A recent study on Ni and Co bioaccessibility in several alloy and metal powders, and simple metal powder mixtures, in gastric fluid, interstitial fluid, and ALF, resulted in similar conclusions as this study (Heim *et al.*, 2020). It was shown that the Ni and Co bioaccessibilities of the alloys can largely differ from those expected from their bulk concentration due to their unique properties as alloys (Heim *et al.*, 2020).

Overall, the results of the investigation of the electrochemical characteristics, surface oxide composition, and extent of metal release of this study clearly elucidate the importance of the passive surface oxide of the alloy powders compared with the metal powders. Further similar investigations could in future be used to screen and group alloys and metals with the aim to refine their hazard assessment.

## Conclusions

This study quantifies the release of Co and Ni from thoroughly characterized alloy powder particles of stainless steel and Inconel of relevance for several occupational settings compared with Co and Ni metal powders. The following main conclusions were drawn:

(1) All powders had comparable particle sizes within the respirable range when immersed in synthetic body fluids. Mn(III/IV)-oxides were strongly enriched within the outermost surface oxide of all stainless steel powders (316L, 430, and 304). Cr(III)-oxides were present on both the stainless steel and the Inconel powders.
(2) A relatively high corrosion resistance was observed for the stainless steel powders in all solutions compared with the Inconel alloy and the metal powders. This was related to their passive surface oxide characteristics governed by the presence of Cr(III)-oxides.
(3) All alloy powders (0.1–64 wt.% Ni bulk content) released in all fluids substantially lower amounts of Ni per powder mass (20–20 000-fold after 4 h; 50–30 000-fold after 168 h) compared with the Ni metal powder. The release of Ni increased with the solution acidity and probably with the complexation capacity. The release of Ni was 4–3000-fold lower for the stainless steel and Inconel alloy powders when compared with the Ni metal powder and when compared with its bulk alloy content.
(4) A 2000–300 000-fold lower Co release per powder mass was observed after 168 h exposure for the alloy powders (0.01–0.07 wt.% Co bulk content) compared with the Co metal powder (2000–3 000 000-fold lower after 4 h). Most Co was released in the acidic fluids ALF and GST. The release of Co from the stainless steel and Inconel alloy powders was for all fluids 1.3–24-fold lower when compared with the Co metal powder and the bulk alloy content. Some precipitation of released Co was evident in artificial saliva, hence underestimating the total amount of released Co.
(5) The importance of investigations of electrochemical characteristics, surface oxide composition, and extent of metal release from metal/alloy powders to generate bioaccessibility data and screen groups of metallic materials is highlighted for an improved and refined hazard assessment that today mostly is based on bulk alloy contents. This is further corroborated by findings on the relative bioaccessibility of released metals from alloys, shown to largely differ from 1.

## Funding

Funding for this project was to a large extent provided by the Chinese Scholarship Council, Beijing, China, and Team Stainless, Brussels, Belgium.

## Conflicts of interest

Team Stainless provided the powders investigated, their composition information (which was confirmed by analysis of the authors), and information on current legislative discussions regarding cobalt. The manuscript’s contents, including any opinions and/or conclusions expressed, are those of the authors alone and do not necessarily reflect the policy of either the Chinese Scholarship Council or Team Stainless.

## Supplementary Material

wxaa042_suppl_Supplementary-Material
