# Supplementary material for: Bioaccessibility of Nickel and Cobalt Released from Occupationally Relevant Alloy and Metal Powders at Simulated Human Exposure Scenarios
Source: Ann Work Expo Health. 2020 Apr 22;64(6):659–75. doi: 10.1093/annweh/wxaa042 (PMC7328476; doi:10.1093/annweh/wxaa042)
Supplement: wxaa042_suppl_Supplementary-Material [file wxaa042_suppl_supplementary-material.pdf]

Supplemental Material

Bioaccessibility of nickel and cobalt released from occupationally relevant alloy and metal powders at simulated human exposure scenarios

Xuying Wang, Inger Odnevall Wallinder, and Yolanda Hedberg

S1. Materials and methods

S1.1 Fluid preparation

The chemical composition (g/L) of each synthetic body fluid used in this study is presented in Table S1. Ultrapure water (resistivity-18.2 MΩcm, Millipore, Sweden) was used as solvent for all fluids. The pH was adjusted to the stipulated level (ASW - pH 6.5, ASL - pH 6.75, ALF - pH 4.5 and GST - pH 1.5) using NaOH (ASW, ASL and ALF), and checked using a pH meter (PHM210 Standard pH Meter, MeterLab®, Radiometer Analytical SAS, France). All vessels and equipment in contact with the solutions and blank samples were acid-cleaned in 10 vol-% HNO_3 for at least 24 h and rinsed at least four times with ultrapure water to avoid any metal contamination.

Table S1. Chemical composition (g/L) of the four synthetic body fluids.

**Chemicals**	**ASL[a] pH 6.75**	**ASW[b] pH 6.5**	**ALF[c] pH 4.5**	**GST[d] pH 1.5**
$MgCl_2$	-	-	0.0497	-
NaCl	0.4	5.0	3.21	-
KCl	1.21	-	-	-
Na_2HPO_4	-	-	0.071	-
Na_2SO_4	-	-	0.039	-
$CaCl_2 \cdot 2H_2O$	-	-	0.128	-
$C_6H_5Na_3O_7 \cdot 2H_2O$ (Na_3 citrate dihydrate)	-	-	0.077	-
NaOH	-	-	6.00	-
$C_6H_8O_7$ (citric acid)	-	-	20.8	-
H_2NCH_2COOH (glycine)	-	-	0.059	-
$C_4H_4O_6Na_2 \cdot 2H_2O$ (Na_2 tartrate dihydrate)	-	-	0.090	-
$C_3H_5NaO_3$ (Na lactate)	-	-	0.085	-
$C_3H_3O_3Na$ (Na pyruvate)	-	-	0.086	-
$(NH_2)_2CO$ (urea)	1.0	1.0	-	-
$NaH_2PO_4 \cdot 2H_2O$	0.78	-	-	-
$Na_2S \cdot 9H_20$	0.005	-	-	-
$CH_3CHOHCO_2H$ (lactic acid)	-	1.0	-	-
25% HCl	-	-	-	4.0

a. Artificial sweat (ASW, according to EN1811).

b. Artificial saliva (ASL).

c. Artificial lysosomal fluid (ALF).

d. Artificial gastric solution (GST).

S1.2 Metal release analysis

For the metal release analysis by means of AAS and GF-AAS, calibration was performed using 1% HNO_3 and three or four metal standards (10, 30, 100 µg/L for Ni, and 10, 30, 60, 100 µg/L for Co in GF-AAS; 1, 3, 10 mg/L for Ni, and 1, 3, 10, 30 mg/L for Co in AAS) and repeated if the calibration curve fitting had a correlation coefficient less than 0.995. All analyses were based on triplicate readings of each sample. A quality control sample of known concentration was analysed every 5th solution sample to ensure accurate analysis. The limits of detection were 0.95 µg/L for Ni and 1.2 µg/L for Co. If the concentration of the solution sample was out of calibration range, it was diluted with 1% ultrapure HNO_3 (up to 30 times for Ni and Co analysis from the metal powders) and reanalyzed.

S1.3 Static light scattering

Refractive indexes of the alloys (2.757), Co metal (2.1396), Ni metal (1.958) and water (1.33, as the solvent of all fluids), as well as the particle density [304: 7.8 g/cm^3, 316L: 7.9 g/cm^3, 430: 7.8 g/cm^3, IN625: 8.44 g/cm^3, Ni: 7.33 g/cm^3 (accounting for 20% agglomeration), Co: 4.93 g/cm^3 (accounting for 50% agglomeration)], were used as input parameters to calculate the volume size distribution and particle specific surface area in each test fluid. More details are given in (Wang et al., 2019).

S1.4 Polarization resistance calculation

By plotting the Tafel lines in the polarization curve, the corrosion potential (E_{corr}) and the corrosion current (I_{corr}) can together with the cathodic (b_c) and anodic (b_a) Tafel constants be extracted using the VersaStudio software. The current was not normalized to the surface area, but the exposed PIGE surface area covered by particles was kept as constant as possible (see section 2.4.1. in the main manuscript). The polarization resistance (R_p), a quantitative parameter that can be used to compare the resistance against corrosion under investigated conditions (a high R_p implies a high corrosion resistance), was calculated using Eq. (S1) (Stern and Geary, 1957; Stern, 1958):

$$R_p = \frac{b_c * b_a}{2.3 * (b_c + b_a) * I_{corr}} \qquad eq.\ (S1)$$

S1.5 Cyclic voltammetry measurement

To obtain more detailed information on the oxidation states of the surface oxide metals, cyclic voltammetry (CV) measurements were performed starting at OCP (stabilized for 5 min), polarizing cathodically to approx. -1.4 V and consecutively polarizing anodically to approx. 0.2 V vs. Ag/AgCl saturated KCl (used as reference electrode) at a scan rate of 0.5 mV/s (linearly) in 8M NaOH [described previously in (Chouaib et al., 1981; Linhardt, 1998)] using a µAUTOLAB potentiostat equipped with Nova 3.0 software. The working electrode was prepared, and the choice of reference and counter electrodes is described in section 2.4.1 of the main manuscript. The only difference was that the powder mass attached to the PIGE was weighed (data presented) and adjusted to obtain optimal peak heights in the voltammograms.

S2 Results and discussions

S2.1 XPS spectra of the surface oxides

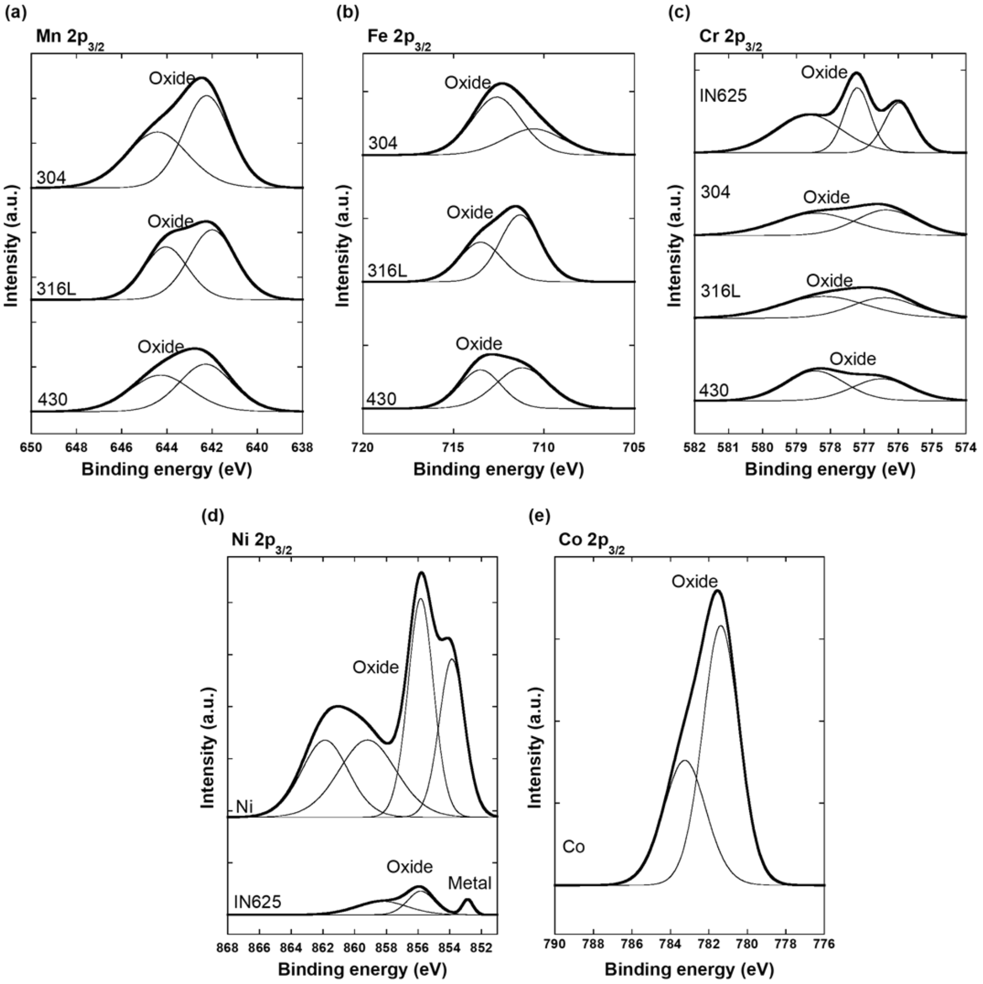

Figure S1. XPS spectra of (a) Mn $2p_{3/2}$ on the stainless steel powders (430, 316L and 304), (b) Fe $2p_{3/2}$ on the stainless steel powders (430, 316L and 304), (c) Cr $2p_{3/2}$ on the stainless steel (430, 316L and 304) and Inconel (IN625) powders, (d) Ni $2p_{3/2}$ on the Inconel (IN625) and Ni metal powders, and (e) Co $2p_{3/2}$ on the Co metal powder, respectively, prior to exposure in synthetic body fluids.

S2.2 Electrochemical surface oxide characterization

Voltammograms of the six powders are presented in Fig. S2. The cathodic polarization curves are marked as dotted lines and the anodic curves as solid lines. In the case of stainless steel powders (304, 316L and 430), Fig. S2a, the first cathodic peak (C1) was assigned to adsorbed oxygen (Chouaib et al., 1981; Hedberg et al., 2012; Hedberg et al., 2013). As previously reported (Chouaib et al., 1981; Hedberg et al., 2012; Hedberg et al., 2013), several Mn (III or IV)-oxides can be correlated to the cathodic peaks at -0.55 V (C2), -0.9 V (C3) and -1.07 V (C4), and their corresponding anodic peaks at -0.4 V (A3) and -0.2 V (A4). The cathodic peak of Fe(III) was not clearly observed as it might have been shadowed by the hydrogen peak. However, the anodic peaks of Fe oxidation were observed at -0.95 V (A2) and -1.1 V (A1) (Chouaib et al., 1981; Linhardt, 1998; Hedberg et al., 2012; Hedberg et al., 2016). The anodic peak at 0.075 V (A5) that corresponds to the oxidation of Cr(III) to Cr(VI) was observed for all stainless steel powders (Hedberg et al., 2012; Hedberg et al., 2016). Voltammograms for the IN625 and the Ni metal powder are presented in Fig. S2b. A peak corresponding to adsorbed oxygen was observed at -0.35 V (C5) for IN625, but not for Ni. The cathodic peaks assigned to the reduction from Ni(II,III) to Ni(0) appeared at -1.0 V (C6) for IN625 and at -0.9 V (C7) for Ni, with the corresponding anodic peaks at -0.85 V (A6) and at -0.8 V (A7), respectively (Beverskog and Puigdomenech, 1997). Even not clearly visible as a peak due to overlap with the oxygen evolution peak, the significantly increased anodic current for IN625 at 0.1 V was attributed to the oxidation of Cr(III), which could explain the more negative reduction potential (C6) related to nickel oxide of the IN625 powder compared to the Ni powder (C7). In the case of the Co metal particles, Fig. S2c, a cathodic peak was observed at -1.0 V (C8), which could correspond to the reduction of Co(II,III) to Co(0) (Chivot et al., 2008). This cathodic peak corresponds to the two broad anodic peaks between -0.9 V and -0.7 V (A8) and between 0 V and 0.2 V (A9) corresponding to the oxidation from Co(0) to Co(II,III) (A8) and from Co(II,III) to Co(III) (A9). In contrast to the other powders, the anodic peaks were much stronger compared to the cathodic peak for the Co metal powder. This suggests that a larger part of the metal was oxidized as compared with its fraction of the initial surface oxide.

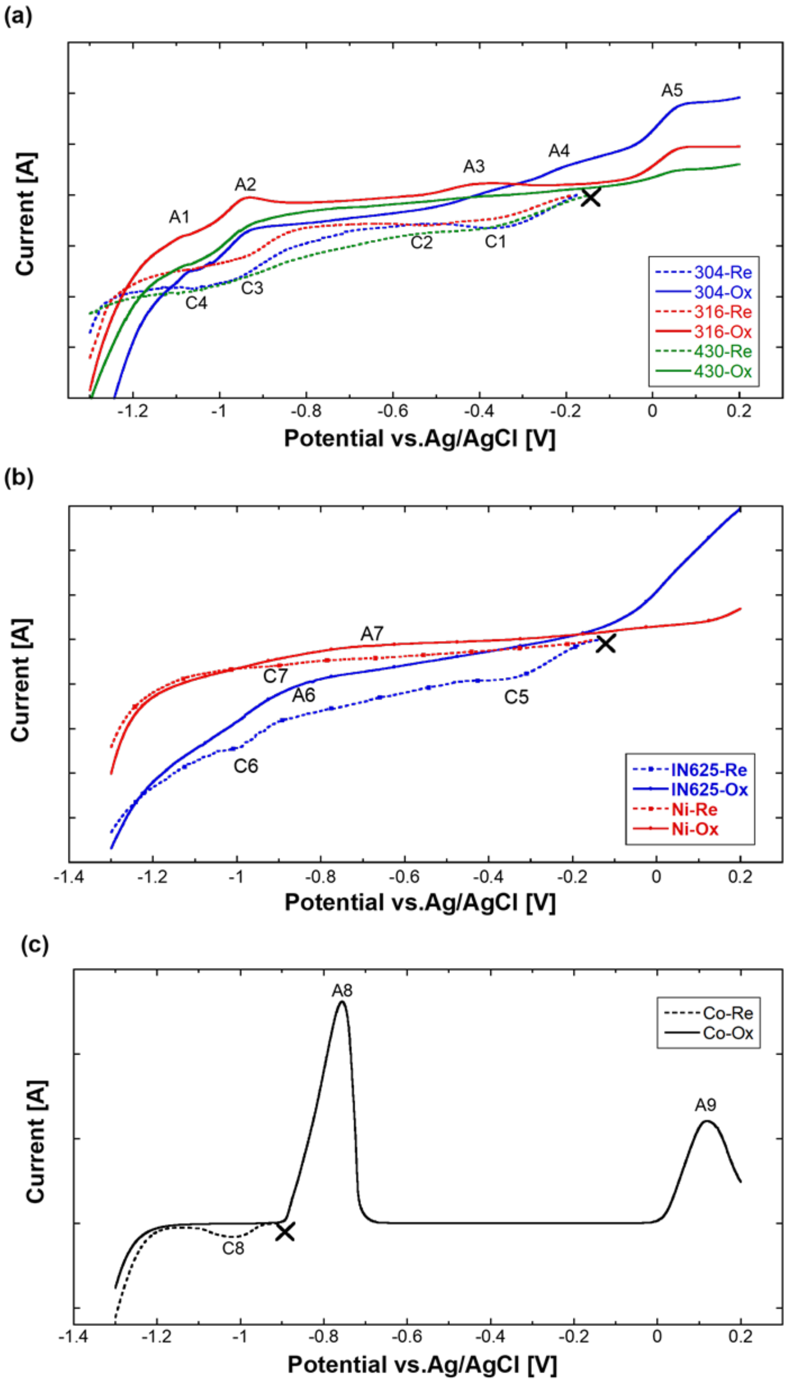

Figure S2. Cyclic voltammograms of (a) the stainless steel (304, 316L and 430) powders, (b) the Ni-based alloy (IN625) and the Ni metal powder, and (c) the Co metal powder, measured by cyclic voltammetry. The particle concentrations on the PIGE surface were 1.68 mg (316L), 6.4 mg (430), 4.36 mg (304), 2.37 mg (IN625), 10.34 mg (Ni) and 3.64 mg (Co), in each case covering a surface area of the PIGE of 0.20 cm^2. A cross marks the OCP (starting point of voltammograms).

S2.3 Parameters of corrosion resistance

Table S2. Corrosion potential (E_{corr}), corrosion current (I_{corr}) and polarization resistance (R_p) of the different powders and test fluids (ASL, ASW, ALF and GST) measured by means of potentiodynamic polarization and recorded using the VersaStudio software. Average data with standard deviation is shown for duplicate independent measurements for each powder. No normalization to surface area was made, which was kept as constant as possible (about 0.20 cm² of powder-covered electrode surface) among measurements and powders.

Solutions	Grade	E_{corr} (mV)	I_{corr} (µA)	R_p (kΩ)
**ASL (pH 6.75)**	***316L***	250±80	0.00021±0.000018	42,000±22,000
	304	170±60	0.00031±0.00011	39,000±36,000
	430	260±200	0.00045±0.00015	35,000±18,000
	IN625	110±120	0.00029±0.00020	100,000±80,000
	Ni	-17±27	0.017±0.00084	1,200±420
	Co	-100±26	0.26±0.048	96±5.8
**ASW (pH 6.5)**	***316L***	240±33	0.00021±0.000049	52,000±33,000
	304	300±40	0.00025±0.0000043	56,000±8,000
	430	230±0.16	0.00026±0.000042	31,000±6,100
	IN625	78±17	0.00028±0.00016	91,000±58,000
	Ni	-1.8±6.4	0.025±0.0074	770±520
	Co	-5.3±33	0.071±0.055	450±270
**ALF (pH 4.5)**	***316L***	430±5.3	0.021±0.0022	1,100±66
	304	430±40	0.013±0.0028	1,200±190
	430	280±130	0.010±0.0040	1,900±140
	IN625	65±15	0.022±0.0036	910±230
	Ni	36±80	0.020±0.0092	1,700±840
	Co	-450±4.1	14±1.3	1.5±0.079
**GST (pH 1.5)**	***316L***	580±47	0.037±0.0015	620±4.90
	304	550±31	0.031±0.0087	640±260
	430	430±150	0.024±0.0034	1,000±250
	IN625	90±20	0.054±0.0046	1,000±210
	Ni	-9.5±9.5	0.16±0.40	130±64
	Co	-410±4.0	160±2.80	0.20±0.0086

S2.4 Bioelution raw data

Table S3. *Released amounts of Ni and release rates of Ni for different powders into ASL (pH 6.75) after different periods of exposure. Mean values and standard deviations of triplicate samples are shown with the corresponding blank sample subtracted.*

Solution	Grade	Exposure time (h)	Released amount of Ni (µg/g)	Release rate of Ni (µg/g/h)
ASL (pH 6.75)	316L	2	10±6.8	5.1±3.4
		4	20±29	4.9±7.4
		8	0.5±0.9	0.07±0.12
		24	4.6±8.0	0.19±0.33
		168	2.0±0.19	0.12±0.0011
	304	4	3.1±2.7	0.77±0.66
		168	21±17.8	0.12±0.11
	430	4	1.2±1.7	0.3±0.4
		168	11±12	0.064±0.073
	IN625	4	215±60	54±15
		168	322±12	1.9±0.069
	Ni	4	23,098±2,070	5,775±517
		168	58,473±3,307	348±20
	Co	4	6.4±4.9	1.6±1.2
		168	<LOD	<LOD

Table S4. *Released amounts of Ni and release rates of Ni for different powders into ASW (pH 6.5) after different periods of exposure. Mean values and standard deviations of triplicate samples are shown with the corresponding blank sample subtracted.*

Solution	Grade	Exposure time (h)	Released amount of Ni (µg/g)	Release rate of Ni (µg/g/h)
ASW (pH 6.5)	316L	2	<LOD	<LOD
		4	<LOD	<LOD
		8	<LOD	<LOD
		24	<LOD	<LOD
		168	30±8.9	0.18±0.053
	304	4	<LOD	<LOD
		168	8.5±4.7	0.050±0.028
	430	4	1.2±2.0	0.29±0.51
		168	0.77±1.3	0.0046±0.0079
	IN625	4	119±9.7	30±2.4
		168	303±47	1.8±0.28
	Ni	4	2,308±300	577±75
		168	14,362±1,404	85±8.4
	Co	4	<LOD	<LOD
		168	<LOD	<LOD

Table S5. *Released amounts of Ni and release rates of Ni for different powders into ALF (pH 4.5) after different periods of exposure. Mean values and standard deviations of triplicate samples are shown with the corresponding blank sample subtracted.*

**Solution**	**Grade**	**Exposure time (h)**	**Released amount of Ni (µg/g)**	**Release rate of Ni (µg/g/h)**
ALF (pH 4.5)	316L	2	5.0±5.1	2.5±2.6
		4	142±49	36±12
		8	445±57	56±7.2
		24	534±15	22±0.62
		168	415±3.2	2.5±0.019
	304	4	48±11	12±2.8
		168	120±29	0.71±0.17
	430	4	32±56	8.0±14
		168	110±61	0.65±0.36
	IN625	4	252±14	63±3.6
		168	340±19	2.0±0.11
	Ni	4	9,133±896	2,283±224
		168	541,565±45,045	3,223±268
	Co	4	16±16	4.1±4.0
		168	48±31	0.29±0.18

***Table S6**. Released amounts of Ni and release rates of Ni for different powders into GST (pH 1.5) after different periods of exposure. Mean values and standard deviations of triplicate samples are shown with the corresponding blank sample subtracted.*

**Solution**	**Grade**	**Exposure time (h)**	**Released amount of Ni (µg/g)**	**Release rate of Ni (µg/g/h)**
GST (pH 1.5)	316L	2	392±43	196±22
		4	756±32	189±7.9
		8	720±58	90±7.3
		24	746±51	31±2.1
		168	795±22	4.7±0.13
	304	4	266±4.1	66±1.0
		168	345±23	2.1±0.14
	430	4	242±55	61±14
		168	242±16	1.4±0.098
	IN625	4	637±107	159±27
		168	683±42	4.1±0.25
	Ni	4	181,429±18,852	45,357±4,713
		168	888,091±31,369	5,286±187
	Co	4	14±13	3.6±3.2
		168	65±16	0.38±0.097

Table S7. *Released amounts of Co and release rates of Co for different powders into ASL (pH 6.75) after different periods of exposure. Mean values and standard deviations of triplicate samples are shown with the corresponding blank sample subtracted.*

**Solution**	**Grade**	**Exposure time (h)**	**Released amount of Co (µg/g)**	**Release rate of Co (µg/g/h)**
ASL (pH 6.75)	316L	2	1.6±2.5	0.78±1.3
		4	5.8±10	1.4±2.5
		8	14±16	1.7±2.0
		24	<LOD	<LOD
		168	3.9±6.8	0.023±0.041
	304	4	0.064±0.11	0.016±0.028
		168	<LOD	<LOD
	430	4	1.9±1.8	0.47±0.46
		168	<LOD	<LOD
	IN625	4	5.6±2.6	1.4±0.64
		168	15±0.67	0.090±0.0040
	Ni	4	0.53±0.92	0.13±0.23
		168	<LOD	<LOD
	Co	4	200,041±33,724	50,010±8,431
		168	34,794±4,827	207±29

Table S8. *Released amounts of Co and release rates of Co for different powders into ASW (pH 6.5) after different periods of exposure. Mean values and standard deviations of triplicate samples are shown with the corresponding blank sample subtracted.*

**Solution**	**Grade**	**Exposure time (h)**	**Released amount of Co (µg/g)**	**Release rate of Co (µg/g/h)**
ASW (pH 6.5)	316L	2		
		4		
		8		
		24		
		168	0.19±0.33	0.0011±0.0020
	304	4	15±17	3.7±4.2
		168	<LOD	<LOD
	430	4	7.3±13	1.8±3.2
		168	<LOD	<LOD
	IN625	4	<LOD	<LOD
		168	17±6.9	0.10±0.041
	Ni	4	3.6±6.1	0.91±1.5
		168	4.4±3.8	0.026±0.023
	Co	4	27,071±5,094	6,768±1,274
		168	266,265±12,299	1,585±73

Table S9. *Released amounts of Co and release rates of Co for different powders into ALF (pH 4.5) after different periods of exposure. Mean values and standard deviations of triplicate samples are shown with the corresponding blank sample subtracted.*

Solution	Grade	Exposure time (h)	Released amount of Co (µg/g)	Release rate of Co (µg/g/h)
ALF (pH 4.5)	316L	2	0.27±0.32	0.14±0.16
		4	64±32	16±7.9
		8	221±13	28±1.7
		24	250±14	10±0.60
		168	170±8.6	1.0±0.051
	304	4	6.5±3.0	1.6±0.75
		168	40±16	0.24±0.094
	430	4	18±4.3	4.5±1.1
		168	27±5.8	0.16±0.035
	IN625	4	26±2.1	6.5±0.54
		168	32±2.1	0.19±0.013
	Ni	4	0.48±0.83	0.12±0.21
		168	11±5.7	0.066±0.034
	Co	4	313,921±35,466	78,480±8,867
		168	1,102,700±267,470	6,560±1,590

Table S10*. Released amounts of Co and release rates of Co for different powders into GST (pH 1.5) after different periods of exposure. Mean values and standard deviations of triplicate samples are shown with the corresponding blank sample subtracted.*

**Solution**	**Grade**	**Exposure time (h)**	**Released amount of Co (µg/g)**	**Release rate of Co (µg/g/h)**
GST (pH 1.5)	316L	2	101±90	51±45
		4	322±3.9	80±0.97
		8	330±46	41±5.8
		24	366±30	15±1.3
		168	369±30	2.2±0.18
	304	4	55±5.8	14±1.5
		168	53±3.2	0.32±0.019
	430	4	21±19	5.3±4.7
		168	54±3.2	0.32±0.019
	IN625	4	4.6±7.9	1.1±2.0
		168	94±8.6	0.56±0.051
	Ni	4	3.1±1.5	0.77±0.37
		168	20±3.8	0.12±0.023
	Co	4	772,163±57,613	193,040±14,403
		168	1,057,700±198,060	6,300±1,180

S2.5 Relative bioaccessibility of Ni and Co after 4 h of exposure

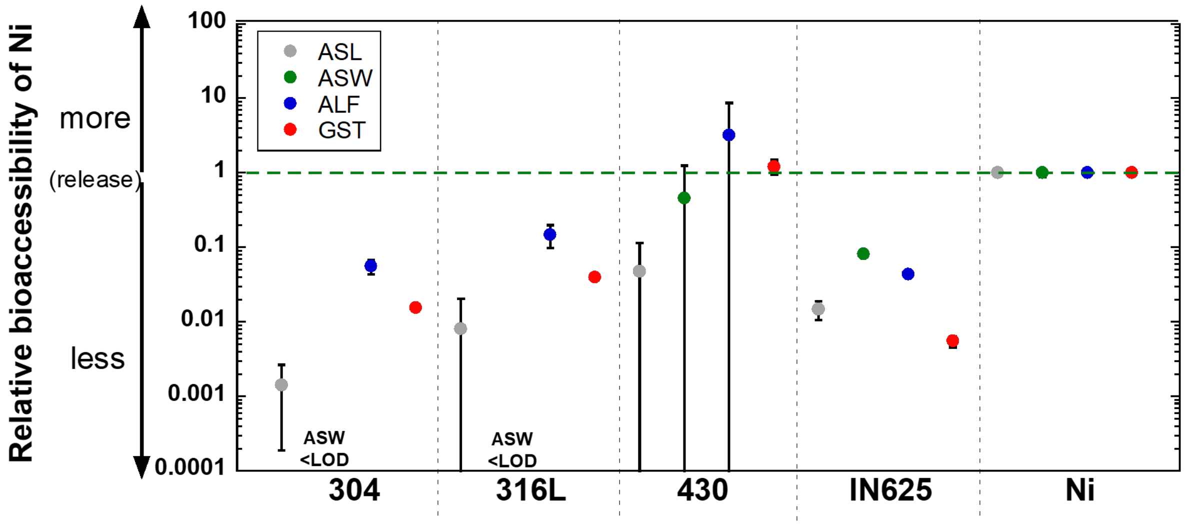

Figure S3. Calculated relative bioaccessibility of Ni released from all alloy- and metal powders exposed at parallel conditions for 4 h. For example, a value of 0.01 means 100 times lower release of Ni per Ni alloy content (by mass) as compared with the Ni metal powder. Per definition, the relative bioaccessibility of the Ni metal powder equals 1, Eq. (1) (main manuscript).

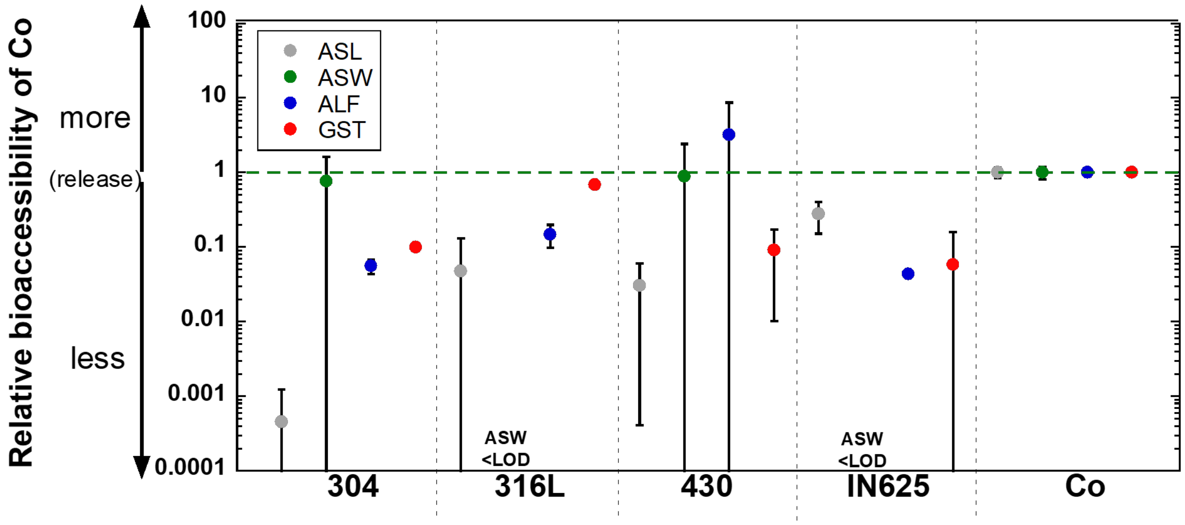

Figure S4. Calculated relative bioaccessibility of Co released from all alloy- and metal powders exposed at parallel conditions for 4 h. For example, a value of 0.01 means 100 times lower release of Co per Co alloy content (by mass) as compared with the Co metal powder. Per definition, the relative bioaccessibility of the Co metal powder equals 1, Eq. (1).

References

Beverskog B, Puigdomenech I. (1997) Revised Pourbaix diagrams for nickel at 25–300 C. Corros Sci; 39: 969-80.

Chivot J, Mendoza L, Mansour C, Pauporté T, Cassir M. (2008) New insight in the behaviour of Co–H2O system at 25–150 C, based on revised Pourbaix diagrams. Corros Sci; 50: 62-69.

Chouaib F, Cauquil O, Lamache M. (1981) Comportement electrochimique d'oxydes de manganese, en milieu alcalin. Electrochim Acta; 26: 325-28.

Hedberg Y, Norell M, Hedberg J, Szakálos P, Linhardt P, Odnevall Wallinder I. (2013) Surface characterisation of fine inert gas and water atomised stainless steel 316L powders: formation of thermodynamically unstable surface oxide phases. Powder Metall; 56: 158-63.

Hedberg Y, Norell M, Linhardt P, Bergqvist H, Odnevall Wallinder I. (2012) Influence of surface oxide characteristics and speciation on corrosion, electrochemical properties and metal release of atomized 316L stainless steel powders. Int J Electrochem Sc; 7: 11655-77.

Hedberg YS, Herting G, Latvala S, Elihn K, Karlsson HL, Odnevall Wallinder I. (2016) Surface passivity largely governs the bioaccessibility of nickel-based powder particles at human exposure conditions. Regul Toxicol Pharmacol; 81: 162-70.

Linhardt P. (1998) Electrochemical identification of higher oxides of manganese in corrosion relevant deposits formed by microorganisms. Trans Tech Publ; 289: 1267-1274.

Stern M. (1958) A method for determining corrosion rates from linear polarization data. Corrosion; 14: 60-64.

Stern M, Geary AL. (1957) Electrochemical polarization I. A theoretical analysis of the shape of polarization curves. J Electrochem Soc; 104: 56-63.

Wang X, Herting G, Wei Z, Odnevall Wallinder I, Hedberg Y. (2019) Bioaccessibility of nickel and cobalt in powders and massive forms of stainless steel, nickel-or cobalt-based alloys, and nickel and cobalt metals in artificial sweat. Regul Toxicol Pharmacol; 106: 15-26.
